# The Reinforcing Effect of Cluster Materials in the Combustion of Hydrocarbon Fuels

**DOI:** 10.3390/ijms27104374

**Published:** 2026-05-14

**Authors:** Xiao Wang, Xiaogang Mu, Yue Zhang, Shenghui Wang, Rui Wang, Junda Wang

**Affiliations:** Zhijian Lab, Rocket Force University of Engineering, Xi’an 710025, China; wangxiao1025921@163.com (X.W.); wshalano@163.com (S.W.); violet_wr1994@163.com (R.W.); 18055852903@163.com (J.W.)

**Keywords:** hydrocarbon fuel combustion, cluster materials, cluster stability, combustion enhancement, fuel system compatibility

## Abstract

Hydrocarbon fuels are a vital component of the global energy supply, owing to their excellent energy density and high burnability. It has been demonstrated that the addition of atomically precise cluster materials to hydrocarbon fuels as additives is a promising approach to achieve breakthroughs in improving their combustion performance. Though cluster materials show great potential in boosting combustion performance, their large-scale synthesis, insufficient thermochemical stability, agglomeration and deactivation have constrained their practical applications. Hence, researchers have adopted strategies such as ligand-engineered stabilization, carrier-confined encapsulation, in situ synthesis and surface functionalization to enhance their stability and dispersion in complex combustion environments. Meanwhile, studies on the compatibility of cluster materials with hydrocarbon fuels have also played a crucial role in evaluating the engineering feasibility of cluster materials, including their dissolution and dispersion behavior, interfacial interactions, and long-term storage stability. With regard to performance enhancement, it has been demonstrated through numerous studies that the addition of clusters can have a massive impact on combustion efficiency, thermal stability and ignition performance. This article reviews the ways cluster materials can improve combustion performance via molecular design and synergistic effects, extending the existing research.

## 1. Introduction

Due to their exceptional advantages in the field of propellants, high-energy-density hydrocarbon fuels have been a hot topic of research in the aerospace sector [1]. Hydrocarbon fuels can provide rocket engines and ramjet engines with greater thrust and specific impulse, thereby enabling aircraft to carry heavier payloads, reach higher speeds, or extend their range [2]. Although hydrocarbon fuels inherently possess good combustion properties, their combustion processes still encounter challenges under specific conditions, such as supersonic combustion, micro-scale combustion, or low-temperature ignition. The above problems mainly appear as long ignition delay time, low combustion efficiency, and high levels of polycyclic aromatic hydrocarbons (PAHs) and soot [3,4]. These problems not only impede further improvements in energy conversion efficiency but also aggravate environmental pollution. Therefore, the development of a new additive in this fuel has become a research hotspot. Hence, the application of additives could improve the combustion performance of hydrocarbon fuels and reduce emissions of pollutants.

In recent years, metal nanoclusters (NCs) and ultrasmall nanoparticles (NPs) with precise atomic numbers have attracted widespread attention in fields such as electrocatalysis owing to their unique geometric and electronic structures, as well as their size characteristics, which lie between those of single-atom catalysts and nanoparticles [5,6]. Nanocluster materials consist of a few to several hundred atoms and possess discrete energy levels, high specific surface areas, and tunable electronic structures; it is precisely these characteristics that enable them to exhibit excellent activity and selectivity in catalytic reactions [7]. If such nanocluster materials are used as fuel additives, they are expected to significantly improve the combustion properties of hydrocarbon fuels in several ways, such as by lowering activation energy, promoting radical chain reactions, and accelerating the oxidation of intermediates, thereby enhancing ignition performance, the flame propagation rate, and the heat release rate [7].

However, although nanocluster materials have broad theoretical application prospects, their practical application in hydrocarbon fuel systems still faces numerous challenges [5]. On the one hand, the controlled, large-scale synthesis of high-purity, monodisperse nanoclusters with well-defined crystalline phases remains a technical challenge [7]. On the other hand, in complex hydrocarbon media, nanoclusters are prone to agglomeration, oxidation, or ligand dissociation, leading to a decline in catalytic activity and insufficient long-term stability [5,8,9]. In response to these challenges, researchers are actively exploring various strategies, including ligand engineering, surface silanization, and in situ stabilization methods such as confined encapsulation, which can enhance the stability of cluster materials in fuel environments and extend their catalytic lifespan [5,7,8].

Furthermore, a key prerequisite for the rational design of high-performance fuel additives lies in a deep understanding of the interfacial compatibility between cluster materials and hydrocarbon fuels, as well as the precise regulation of their interactions [10]. Once the preparation and stability of cluster materials are resolved, and their compatibility with hydrocarbon fuels is further optimized, cluster materials are expected to significantly enhance the combustion performance of hydrocarbon fuels. This performance enhancement is primarily reflected in improved combustion efficiency, enhanced thermal stability, improved ignition performance, and reduced ignition delay time [3,11].

This review aims to systematically explore the performance of cluster materials in enhancing the combustion of hydrocarbon fuels, including their mechanisms of action, pathways for improving stability, design principles for compatibility, and the patterns of their influence on key combustion performance parameters. We expect to provide a theoretical foundation and technical reference for the rational design of high-performance fuel additives through a systematic review and analysis of the literature.

## 2. Combustion Properties of Hydrocarbon Fuels

### 2.1. Structure–Activity Relationships in the Molecular Structure and Combustion Properties of Hydrocarbon Fuels

In recent years, as high-precision experiments, quantum chemical calculations, and machine learning technologies have converged, researchers’ understanding of the relationship between the molecular structure of hydrocarbon fuels and their combustion properties has gradually shifted from qualitative descriptions to a new phase of quantitative prediction. Key elements of molecular structure include carbon chain length, degree of branching, types of saturated bonds, cyclic structures such as aliphatic and aromatic rings, and molecular structural features such as the types of functional groups and their positions. Together, these characteristics are the decision-makers of key combustion properties such as ignition delay time (IDT), laminar flame speed, calorific value, and pollutant formation trends such as soot and nitrogen oxides.

Straight-chain alkanes, with their regular molecular structure and lower steric hindrance, generally show higher reactivity, such as shorter IDT and higher laminar flame speeds. Peng et al.’s [12] research on n-pentane (n-C_5_H_12_) showed that its IDT was far shorter than those of highly branched isomers with the same number of carbon atoms under high temperature. This difference is due to the fact that branching increases the intramolecular steric hindrance, which inhibits the attack of key radicals such as ·OH on C-H bonds, thereby reducing the low-temperature oxidation performance and prolonging the delay time [13]. However, Liang et al. [14] argued that the presence of unsaturated C=C double bonds in the alkene molecules made the π-electron cloud more easily attacked by radicals. Therefore, it usually has higher reactivity at low temperature than its corresponding alkane, which in turn directly affects low-temperature combustion and knock in engines.

The effect of ring structures on fuel combustion performance is even more complex. Small cycloalkanes, such as cyclopropane, exhibit significant ring strain and extremely high reactivity, resulting in a significantly shorter ignition delay [15]. In contrast, large cycloalkanes, such as cyclohexane, demonstrate greater chemical stability. When the ring structure becomes an aromatic ring, the combustion characteristics undergo a fundamental change. Benzene and its derivatives, due to their high resonance stabilization energy and C-H bond dissociation energy, are more difficult to ignite, exhibit lower laminar flame speeds, and readily generate PAHs and soot precursors through mechanisms such as hydrogen abstraction–acetylene addition [16,17]. Liu et al. [18] experimentally demonstrated that, under identical conditions, the laminar flame speed of benzene is significantly lower than that of cyclohexane and cyclohexene, while the latter two are in turn lower than that of straight-chain alkanes, clearly illustrating the decisive role of unsaturation and ring type in flame propagation kinetics.

The combustion behavior of hydrocarbon fuels is not an inherent property but an external representation of the underlying molecular structure. For atomic alkane homologues to the complex PAHs, subtle structural variations can systematically control the overall combustion process via the formation of critical radicals, the stability of the peroxide isomer, and the product distribution.

### 2.2. Limitations of the Combustion Properties of Hydrocarbon Fuels

In recent years, research on the combustion properties of traditional hydrocarbon fuels has reached a relatively mature stage, and the underlying structure–activity relationships have been systematically elucidated. As the demand for higher conversion efficiency in propulsion systems, power units, and energy sources continues to grow, the approach of relying solely on optimizing hydrocarbon molecular structures to improve key combustion parameters, such as ignition delay time and laminar flame speed, is increasingly revealing its fundamental limitations at the physicochemical level.

Table 1 compares oxygenated additives, organometallic compounds, nanoparticles, and emerging cluster materials, analyzing them in terms of their mechanisms of action, performance enhancement potential, and practical application prospects. The results indicate that cluster materials, due to their unique physicochemical properties, have been identified as a key performance-enhancing substance.

Recent studies strongly support our conclusion. Zhang et al. [19] developed a new minimum ignition energy (MIE) estimation based on microscopic molecular structure, which is a theoretical criterion to be applied for understanding the influence of any additive on the fuel ignition sensitivity. Kevadiya et al. [20] combined a functional group approach with machine learning and precisely estimated the MIE of 55 organic molecules. This provides a promising route for achieving the precise control of ignition performance at the molecular level. And Chen et al. [21] proposed a “conjoint fingerprints” machine learning framework that can predict multiple physicochemical properties of complex molecules with accuracy (such as R^2^ > 0.9). This framework could be directly applied to build the structure–property relationship model of “hydrocarbon-cluster” composite systems. By encoding the distinct characteristics of clusters (e.g., the number of atoms, geometric structure, electronic structure) as descriptors, the enhancement effect of clusters on combustion performance can be quantitatively predicted.

A systematic comparison with classic additives suggests that cluster materials could be promising substances for addressing bottlenecks in hydrocarbon fuel combustion performance; their advantages are mainly derived from their atomic precision, perfect dispersion stability, highly efficient catalytic activity and controllable energy release behavior.

## 3. Clustered Materials

Although cluster materials have shown great potential in improving hydrocarbon fuel combustion performance, they still face a series of challenges in practical applications, mainly related to difficulties in preparation and insufficient stability.

### 3.1. Stability of Clustered Materials

With the rapid advancement of atomically precise synthesis techniques, in situ characterization methods, and theoretical simulation approaches, researchers’ understanding of cluster stability mechanisms has gradually transitioned from empirical observation to rational design. Stability not only determines whether it can be isolated and characterized under experimental conditions, but also directly affects the performance in catalysis, optoelectronics, and sensing.

#### 3.1.1. Electronic Structure

The “hyperatomic” model and the Jellium model provide a theoretical framework for understanding the “magic number” stability of metal clusters. Yi et al. [22] reported that the ${\mathrm{Nb}}_{15^{-}}$ cluster, which exhibits significant chemical inertness due to its highly ordered hyperatom state, undergoes almost no dehydrogenation during reactions with alkenes due to its closed electron shell and large HOMO-LUMO energy gap. Similarly, Tang et al. [23] reported that the Ga_13_M (M = Li, Na, K, Rb) series of clusters achieves an 18-electron closed-shell structure and exhibits enhanced stability, high vertical ionization potentials, and low electron affinity, which fully aligns with the predictions of the Jellium model. The Wade–Mingos rules and the Polyhedral Skeleton Electron Pair Theory are more suitable for main-group element clusters. Studies have shown that for clusters such as Al_7_M (M = Si, Ge, N, P), stability strongly depends on the effect size of the impure atoms; smaller central atoms can effectively alleviate surface tension and enhance overall stability [24].

**Figure 1:**
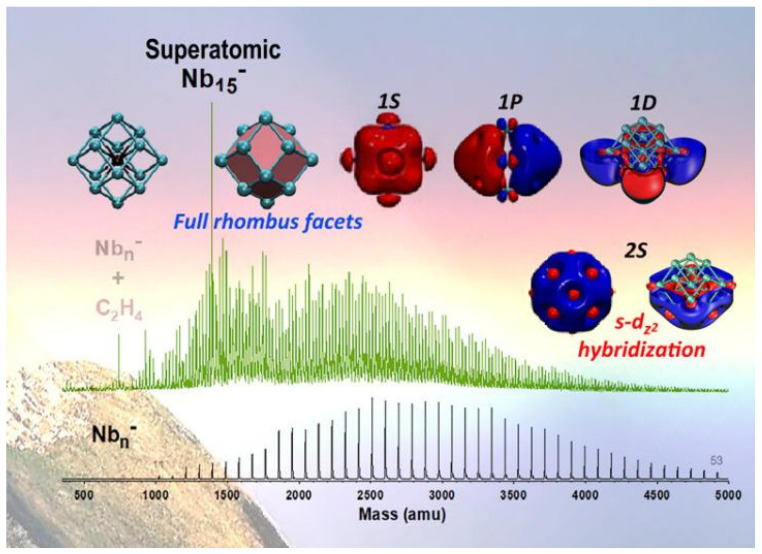
${\mathrm{Nb}}_{15^{-}}$ schematic diagram of the structure and electronic properties of a hyperatom cluster. Reprinted with permission from ref. [22]. © 2023 American Chemical Society. All rights reserved.

Figure 1 primarily illustrates the hyperatomic properties of the ${\mathrm{Nb}}_{15^{-}}$ cluster, including its highly symmetric rhombohedral geometry and unique hyperatomic electron shell structure (1S 1P 1D), and the key s-$d_{z^{2}}$ hybridization within it. Furthermore, based on the mass (amu) and corresponding numerical ranges provided by the mass spectrometry data in the figure, it is evident that the ${\mathrm{Nb}}_{15^{-}}$ cluster exhibits a high abundance under the experimental conditions and demonstrates marked inertness toward alkenes; these two observations further corroborate its exceptional stability as a “double magic number” cluster [22].

#### 3.1.2. Ligand Engineering

Organic ligands such as thiols, phosphines, and alkyne groups, as well as inorganic ligands such as polyoxometalates (POMs), can effectively suppress the Ostwald ripening and sintering processes of clusters by saturating the surface dangling bonds of metal atoms. Recent studies have shown that disrupting open coordination sites can significantly enhance the stability of silver nanoclusters; for example, silver clusters protected by thio-cuparene exhibit stability far exceeding that of traditional closed-shell systems with eight electrons [25]. In addition, POMs have been successfully used as multidentate ligands to stabilize gold–silver alloy nanoclusters, not only conferring excellent thermal stability but also synergistically enhancing their photocatalytic performance [26]. This synergistic interaction between the ligand and the cluster opens up new avenues for designing cluster materials that combine high stability with multifunctionality [27].

**Figure 2:**
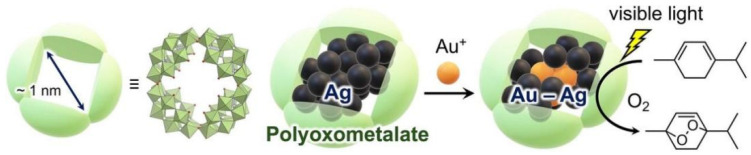
Synthesis of Au-Ag alloy nanoclusters in a ring-shaped POM P8 W48 cavity. Reprinted from ref. [26].

Figure 2 shows the synthesis of surface-exposed Au–Ag alloy nanoclusters within ring-shaped POM by reacting Ag nanoclusters with Au^+^. The obtained Au–Ag alloy nanoclusters display remarkably improved visible-light-responsive photocatalytic ability and stability in aerobic oxidation reactions compared to the Ag NCs parent [26]. Reprinted from ref. [26].

#### 3.1.3. Carrier Interactions

The additional anchoring of clusters to metal–organic frameworks (MOFs) or oxide supports offers the possibility to further improve the cluster stability via utilizing confinement effects and/or strong metallic support interactions. Song et al. [28] discussed the stability of orbitally arranged Pt nanoclusters in MOF-808, where the strong interactions between the clusters and MOF framework nodes effectively prevent cluster movement and aggregation. Similarly, Y-Ti-O nanoclusters in oxide dispersion-strengthened steels confer exceptional creep resistance to the material due to their ultra-high-density distribution within the ferritic matrix, which is attributed to the good lattice matching and interfacial stability between the clusters and the matrix [29].

**Figure 3:**
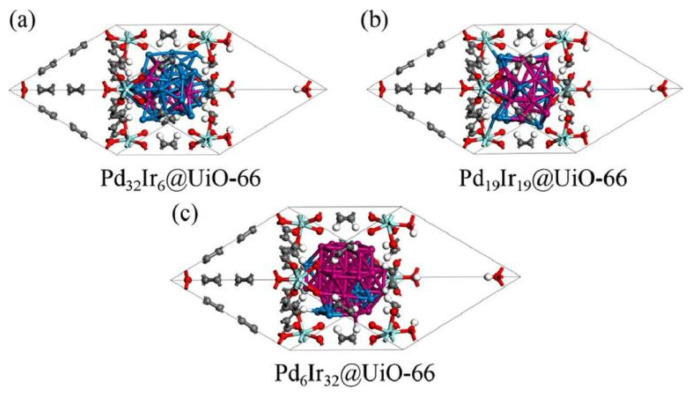
The stable structures of (**a**) Pd_32_Ir_6_, (**b**) Pd_19_Ir_19_ and (**c**) Pd_6_Ir_32_ loaded in UIO-66. Reprinted with permission from ref. [30]. © 2023 Elsevier B.V. All rights reserved.

Figure 3 shows the stable structures formed when three Pd-Ir alloy nanoclusters with different compositions—Pd_32_Ir_6_, Pd_19_Ir_19_, and Pd_6_Ir_32_—are supported on UiO-66 material. As shown in the figure, these alloy clusters undergo deformation and distortion within the octahedral pores of UiO-66, with the structural changes in the Pd_32_Ir_6_ clusters being the most pronounced. This indicates that UiO-66 exerts a significant influence on the structure of Pd-Ir alloy nanoclusters and also helps prevent the agglomeration of metal clusters [30].

#### 3.1.4. Thermal Stability

In Al-Mg-Si(-Cu) alloys, both types of nanoclusters formed via different pathways—Cluster (1) and Cluster (2)—remain stable during a two-step aging process at 250 °C. Furthermore, they serve as effective nucleation sites for the β” phase, significantly enhancing the alloy’s hardness [31]. This finding demonstrates that certain specific clusters indeed possess structural robustness under high-temperature service conditions. By considering a larger set of cluster systems, parameters such as the average binding energy (Eb), second-order energy difference (Δ^2^E) and HOMO-LUMO energy gap are commonly employed to characterize their relative stabilities. Calculations for Ni_n_ (n ≤ 20) clusters indicate that clusters with “magic number” atomic counts (n = 4, 6, 13, 19) exhibit the highest binding and dissociation energies [32]. Similarly, Zhang et al. [33] confirmed the existence of stability peaks at specific sizes through their study on Sn_n_Al_n_ (n = 2–12) clusters.

### 3.2. Techniques for Preparing Clustered Materials

The preparation of clustered materials has been a hot topic in materials science over the past five years, but its complexity and variability have also presented numerous challenges. Clustered materials, which are of sizes ranging from an atom to bulk solids, possess unique electronic, optical, and chemical properties that are the basis of numerous nanotechnologies [34,35,36]. However, due to their unusual size-dependent characteristics, the synthesis of cluster materials is confronted with many challenges, such as atom-scale size control, thermodynamic instability, a tendency to agglomerate, and the absence of a general and versatile preparation strategy.

**Figure 4:**
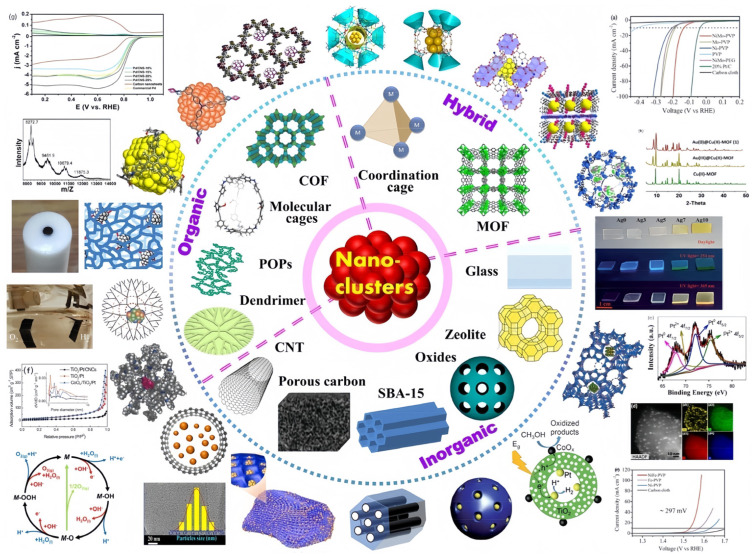
Illustration of MNCs immobilized in various materials, their characterizations and applications in energy catalysis. (**a**) Electrocatalytic linear sweep voltammogram; (**b**) XRD spectrum; (**c**) XPS high-resolution spectrum; (**d**) HAADF-STEM images of nanoclusters/catalysts and the corresponding elemental mapping images; (**e**) The LSV polarization curve of the electrocatalytic reaction; (**f**) Nitrogen adsorption-desorption isotherms and pore size distribution curves; (**g**) Cyclic voltammogram of the electrocatalytic reaction. Reprinted with permission from ref. [37]. © 2023 Elsevier B.V. All rights reserved.

Cluster materials, especially metal nanoclusters (MNCs), have gathered broad attention owing to their unique electronic structure and distinct physicochemical properties. However, a problem lies in the fact that MNCs have small diameters and are prone to agglomeration, while the synthesis process also presents certain challenges; these factors have largely limited their large-scale application. Precisely because of this instability, the preparation of MNCs with precise dimensions and high stability has remained a continuously active research focus in the field of metal nanoclusters [37]. As schematically depicted in Figure 4, MNCs can be immobilized in various materials, followed by systematic characterizations and further applications in energy catalysis. Table 2 systematically summarizes and compares the types, size effects, and combustion properties of different metal clusters, which is of great significance for the rational design of efficient catalysts.

Due to their extremely small size, clustered materials have a very high specific surface area, thereby providing a large number of active sites [50]. This enables them to exhibit excellent performance in catalytic reactions, since catalysis is typically a surface phenomenon; by breaking bulk metal down into tiny clusters, the total surface area available for reactions can be greatly increased [50,51]. Mitchell et al. [7] reported that nanoclusters of precious metals such as platinum, palladium, and rhodium are widely used as catalysts; however, they are prone to sintering at high temperatures, leading to a reduction in active surface area and a decline in catalytic performance.

In addition, theoretical models of cluster compounds are sometimes overly simplified and cannot accurately predict their properties, stability, and functionality. Molecular symmetry can be incorporated into existing cluster models to better study real multi-atom molecules and provide new guidelines for their design [52].

To address these challenges, researchers have developed a variety of innovative strategies over the past five years to improve the monodispersity, stability, reproducibility, and large-scale synthesis capabilities of cluster materials.

#### 3.2.1. Ligand-Assisted Phase Engineering

Ligands play a vital role in the wet chemical synthesis of colloidal nanomaterials. In recent years, it has been found that ligands can regulate the crystalline phase of nanomaterials through ligand-assisted phase engineering, in addition to controlling the size, shape, and stability of nanomaterials. This provides a novel strategy for controlling the structure and properties of nanomaterials. By delivering different ligands, nanocrystals can be guided to crystallize into desired crystalline phases, further tuning the physicochemical properties [53]. This approach is feasible for preparing structurally well-defined and uniform colloidal nanomaterials, and holds great promise for both fundamental research and applications. Nahan et al. [27] in a study in 2025 pointed out that ligand engineering is key in the design of atomically precise metal nanoclusters and cluster assembly. Using the strategies of ligand exchange reaction, structural characterization and stabilization, silver cluster assembly materials with good stability and performance can be prepared, which is very important for maintaining high specific surface area and active sites. The selection of ligands can effectively prevent cluster sintering, thereby preserving their catalytic activity.

#### 3.2.2. Localized Synthesis Strategies

Confining metal clusters within porous nanostructures is an effective method for enhancing their stability and preventing agglomeration. Porous materials such as MOFs and covalent organic frameworks (COFs) are considered as an ideal carrier material due to their high porosity, adjustable pore sizes, and rich functional groups.

MOFs are assembled by linking metal-oxo cluster nodes as secondary building units (SBUs) with organic linkers, providing high structural rigidity, excellent stability, and large surface area [54,55]. Metal clusters encapsulated in the pores of MOFs can effectively suppress sintering and agglomeration of the metal clusters [37]. Noble metal clusters such as Pt and Pd can be encapsulated in the pores of MOFs by this method. Dai et al. used an impregnation-reduction method to introduce Pt precursors into the pore channels of MOF-199, which were subsequently reduced to form Pt nanoclusters. The pore structure of the MOF restricted the growth and migration of the Pt clusters, effectively suppressing sintering and agglomeration at high temperatures, thereby exhibiting higher activity and stability in the catalytic CO oxidation reaction [37,56]. MOFs and their derivatives have shown great potential in fields such as lithium-ion batteries, electrocatalytic redox reactions, and water treatment [56,57,58,59]. Habiba et al. [57] reported that the use of MOF-based materials for the photocatalytic reduction of Cr(VI) represents the latest trend in the field of water purification.

Similar to MOFs, COFs can also serve as carriers for confining clusters. Specifically, for COFs without metal active sites, covalent metal–organic frameworks can be prepared by using metal cluster SBUs derived from MOFs to combine the advantages of the two [55]. It is widely accepted that the organic flux synthesis method is a promising, environmentally friendly, and scalable approach for the synthesis of highly crystalline imine-bonded COFs, which does not suffer from the insufficient crystallinity in the COFs prepared by the traditional solvothermal method [60,61].

**Figure 5:**
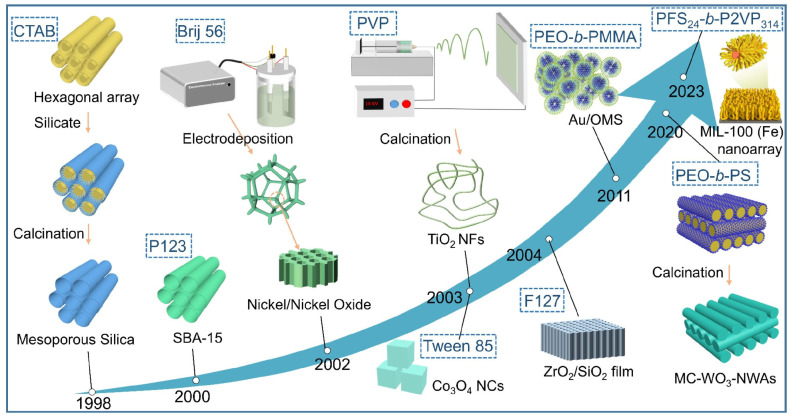
Timeline of the utilization of different non-ionic templates for the synthesis of porous MOS materials. Reprinted with permission from ref. [62]. © 2023 Elsevier B.V. All rights reserved.

Porous metal-oxide-semiconductor (MOS) materials have received widespread attention for gas sensing due to their rich connected pores, abundant active sites, and huge specific surface area. The experimental results have verified that materials prepared using the non-ionic surfactant template method not only provide a good confinement environment for metal clusters, but also regulate the morphology of the metal clusters to achieve optimization of the gas-sensing performance. Figure 5 presents the timeline of the utilization of different non-ionic templates for the synthesis of porous MOS materials. Based on such template strategies, Hu et al. [62] prepared porous SnO_2_ using the template method, and demonstrated that it can provide an appropriate confinement environment for noble metal clusters such as Pt or Pd, which act as sensing elements and modify the gas-sensing performance of SnO_2_. Experimental results showed that the sensitivity and selectivity of gas detection, especially for the detection of toxic gases such as CO and H_2_S, in the porous SnO_2_ loaded with Pd nanoclusters are significantly enhanced. This is due to the effective catalytic effect of the Pd clusters on the adsorption and desorption of target gas molecules.

#### 3.2.3. Precise Synthesis of Hyperatomic Clusters

**Figure 6:**
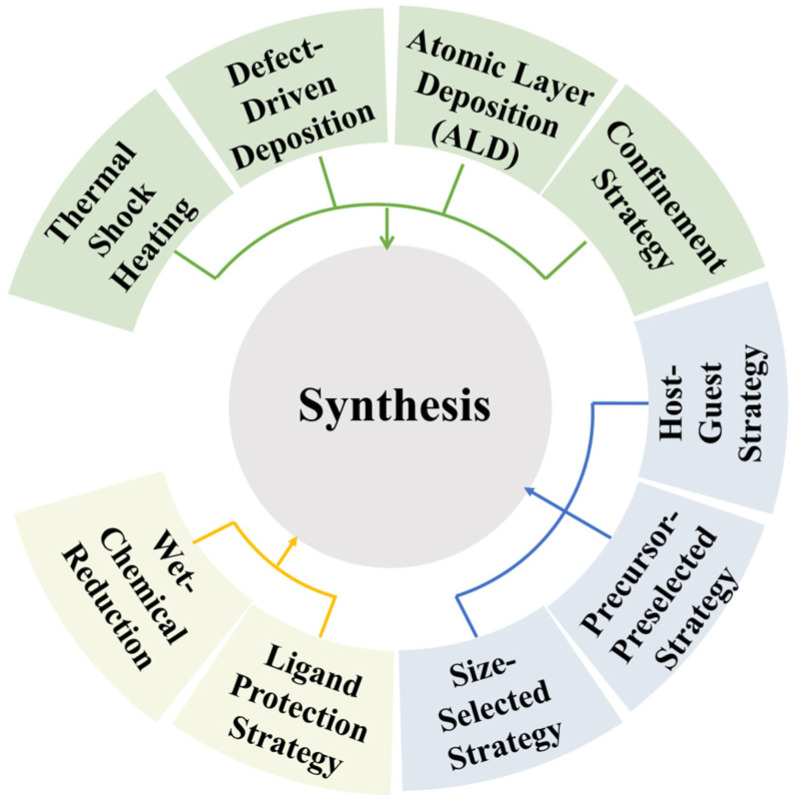
Various nanoclusters and ultrasmall nanoparticle synthesis strategies [5].

In electrocatalytic energy conversion, atomically precise metal nanoclusters and ultrasmall nanoparticles have attracted enormous attention due to their unique geometric and electronic structures. As an intermediate state between single-atom catalysts and nanoparticles, nanoclusters with specific low nuclearity provide designated metal states with multiple atoms or surface sites for the adsorption and transformation of reactants and intermediates. Synthesis strategies and surface/interface engineering are key to achieving highly efficient electrocatalytic performance [5], while Figure 6 illustrates various synthesis strategies for nanoclusters and ultrasmall nanoparticles. Using first-principles calculations, Wang et al. [63] predicted a variety of assembly materials based on Zn_12_O_12_ clusters and investigated their electronic structures and optical properties. By doping these materials with alkaline earth metal atoms, their visible-light absorption properties can be enhanced, providing insights for the design of novel semiconductor materials [64].

#### 3.2.4. Methods for Preparing Clustered Materials

The precise preparation of cluster materials still poses numerous challenges, but several effective synthetic methods have been developed to date. These methods are generally designed to enable precise control over cluster size, morphology, and chemical composition, as well as atomic-level precision. Common methods for preparing cluster materials are shown in Table 3.

As a result of the deepening understanding of the relationship between cluster structure and properties, coupled with the development of intelligent structure-search methods such as computational simulations and machine learning [76,77], it is expected that cluster materials will be designed and prepared more efficiently in the future.

### 3.3. Characterization Methods of Clusters

After the preparation of the cluster materials, generally, characterization methods are used to determine the phases of the products. Additionally, it is necessary to systematically analyze the material’s core properties, including crystal structure, chemical composition, particle size distribution, microstructure, and electronic structure. Precise measurements can not only reveal the key relationship between the structure and the properties of the cluster materials, but also play an indispensable role in the targeted improvement of material performance [78,79].

## 4. Compatibility and Regulation Strategies of Cluster Materials with Hydrocarbon Fuels

Clustered materials could enable the utilization of hydrocarbon fuels in cases where combustion efficiency is of critical importance. However, the compatibility between clustered materials and hydrocarbon fuels is a necessary condition for the practical application of cluster materials [73,80]. Compatibility between cluster materials and hydrocarbon fuels is derived from thermodynamic compatibility, dispersion stability, and interfacial interaction. The combination of all three factors determines whether cluster materials can be used as additives for hydrocarbon fuels with long-term stability.

### 4.1. Compatibility of Cluster Materials with Hydrocarbon Fuels

#### 4.1.1. Thermodynamic Compatibility

The stability of cluster materials in hydrocarbon fuels is of significant importance due to their high energies. If clusters undergo spontaneous decomposition or side reactions in the fuel, this will lead to their failure and may even result in the formation of harmful byproducts [81]. Research by Wang et al. [82] indicates that although high-energy copper hydride clusters exhibit excellent combustion performance, their instability and strong reducing properties hinder their widespread application. As high-calorific-value additives, the stability, density, and energy characteristics of boron-carbon derivatives must be compatible with the fuel system [83].

#### 4.1.2. Dispersion Stability

Hydrocarbon fuels are typically nonpolar media. A key challenge that currently needs to be addressed is how to effectively disperse metal or inorganic cluster materials in nonpolar hydrocarbon media while preventing agglomeration and sedimentation. Aggregation of cluster materials causes the loss of their effective surface area, resulting in the loss of catalytic activity and energy release capability, and may even clog the fuel system. Küçükosman et al. [84] investigated the influence of different metal oxides, Fe_2_O_3_, Fe_3_O_4_, and LaFeO_3_, on the combustion performance of fuels by loading them onto the fuel. The study found that the particle size of the cluster material could affect whether it would sediment in the fuel. They proposed a silane-layer spatial control method to further significantly improve the dispersion stability of boron nanoparticles in hydrocarbon fuels. By controlling hydrolysis and condensation reactions, this method forms a uniform coating on the particle surface, thereby improving combustion efficiency [85].

#### 4.1.3. Interface Interaction

The degree of compatibility between the surface ligands or coating layers of cluster materials and hydrocarbon fuel molecules directly influences the dispersion and behavior of the clusters within the fuel. Ligands must not only protect the cluster core but also facilitate sufficient contact between the clusters and the fuel, thereby influencing the combustion process. Improving the compatibility between ligands and hydrocarbon chains is central to enhancing dispersion stability. Ma et al. [86] proposed that the interfacial durability of the catalyst layer in fuel cells is a key factor determining fuel cell performance and lifespan, a principle closely related to interfacial interactions between materials.

### 4.2. Strategies to Improve the Compatibility of Cluster Materials with Hydrocarbon Fuels

#### 4.2.1. Surface Ligand Engineering

Surface engineering of long-chain alkyl ligands showed a great effect on the solubility and dispersion stability of cluster materials in nonpolar solvents such as hydrocarbon fuels. The strong van der Waals interaction between the long-chain alkyl group and the hydrocarbon fuel molecules can significantly reduce the interfacial energy and suppress the aggregation of the cluster materials. Yamashita et al. [87] demonstrated through studies on the dispersibility of phosphate-modified TiO_2_ and ZrO_2_ nanoparticles in hydrophobic solvents that ligand structure and surface coverage have a significant impact on dispersibility behavior.

**Figure 7:**
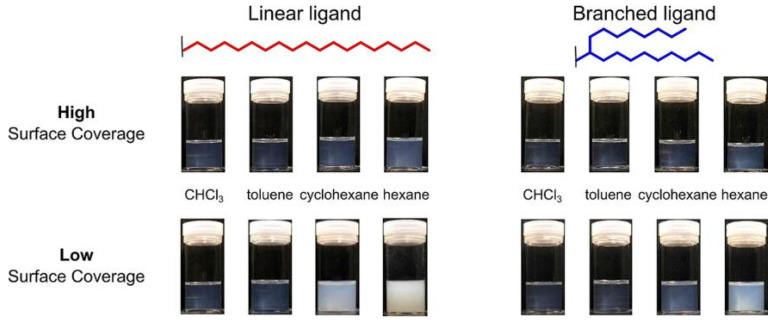
Comparison of surface coverage of linear and branched ligands in different solvents. Reprinted with permission from ref. [87]. © 2023 Elsevier B.V. All rights reserved.

As shown in Figure 7, in the four solvents—CHCl_3_, toluene, cyclohexane, and hexane—linear ligands exhibit high surface coverage, whereas branched ligands generally exhibit lower coverage. This difference suggests that, due to greater steric hindrance, branched ligands cannot pack as tightly as linear ligands, thereby forming a looser ligand layer on the nanoparticle surface; this is precisely the key factor by which they act as “entropy ligands” to enhance the dispersibility of nanoparticles in hydrophobic solvents [87].

Ligands with hydrophobic functional groups can form a protective layer on the surface of the clusters, effectively blocking adverse interactions between the clusters and fuel molecules and reducing cluster aggregation. The surface ligands of gold nanoclusters (Au_13_) can be tailored through a robust amide coupling procedure. Although primarily used in biomedicine, this precise modification method provides a reference for compatibility design in hydrocarbon fuels [88].

**Figure 8:**
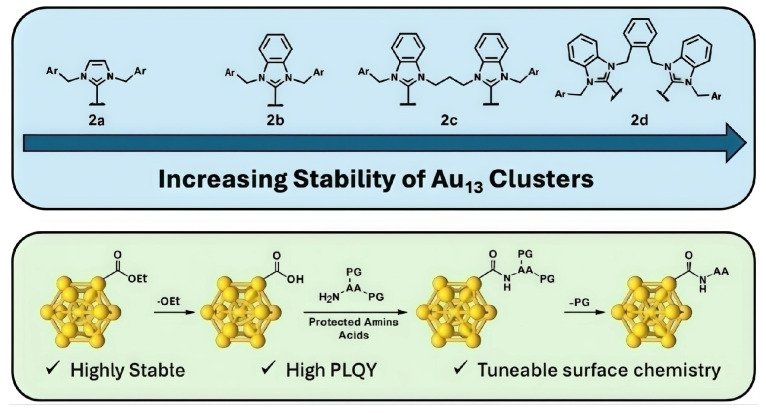
Comparison of surface coverage of linear and branched ligands in different solvents. Reprinted from ref. [88].

Figure 8 illustrates the structural evolution of two groups of Au_13_ nanoclusters. This visual representation demonstrates the impact of different protective ligands on cluster stability. In particular, the Au_13_ clusters show excellent stability between acidic- and base-sensitive protection groups such as Fmoc and OMe, respectively, whereas they show weak stability between acid-sensitive protection groups such as Boc and OBu. The comparison of stability between different protection groups provides a strong experimental basis for the monodisperse product to demonstrate the feasibility of the precise molecular surface modification of atomically precise clusters [88].

#### 4.2.2. Structural Design and Core–Shell Strategy

**Figure 9:**
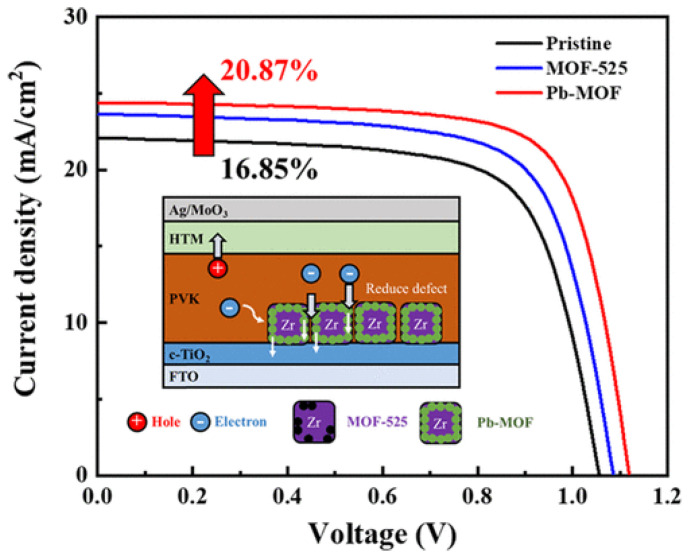
Comparison of surface coverage of linear and branched ligands in different solvents. Reprinted with permission from ref. [89]. © 2023 American Chemical Society. All rights reserved.

The stability of the clusters under harsh conditions can be improved by controlling their geometric and electronic structures, and by considering a core–shell structure. Liu et al. [89] reported that Pb-MOFs modified through post-metallation can serve as a scaffold layer to promote the growth of perovskite crystals, thereby improving stability and crystallinity, as illustrated in Figure 9, which compares the surface coverage of linear and branched ligands in different solvents. The precise atomic structure and functionalization strategies of copper hydride clusters are crucial for their functional properties [90].

By post-metallizing MOF-525 to synthesize a Pb-MOF, it was used as a scaffold between the TiO_2_ layer and the perovskite layer. The Pb-MOF acts as a perovskite nucleation site, facilitating perovskite crystallization, greatly increasing grain size and reducing defects, and improving charge separation. The Pb-MOF-based perovskite solar cell has a conversion efficiency of 20.87%, which retains 86% of the initial efficiency after 40 d. This exceeds both the 16.85% conversion efficiency of the pure PSC (which retains 52% of the initial efficiency after 40 d) and the 18.61% conversion efficiency of the MOF-525 device (which retains 76% of the initial efficiency after 40 d) [89].

Researchers have performed theoretical research, synthesis, and combustion performance verification, and established a comprehensive research framework, thus laying the foundation for the development of high-performance additives for cluster fuels.

## 5. Performance Enhancement of Clustered Materials in Hydrocarbon Fuels

Hydrocarbon fuels are widely used in the aerospace sector due to their high energy density and ease of storage and transportation. Conducting an in-depth comparison of the properties of various cluster materials and investigating the mechanisms by which clusters enhance these properties is of great significance for the development of new, high-efficiency fuels.

### 5.1. Comparison of the Properties of Typical Hydrocarbon Fuels

**Figure 10:**
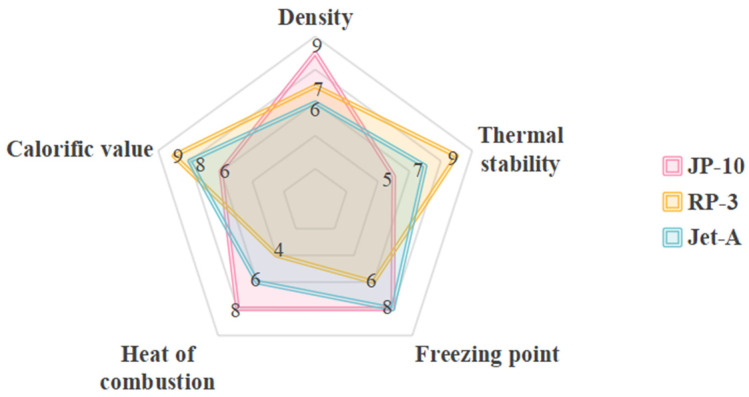
Comparison chart of JP-10, RP-3, and Jet-A performance.

JP-10, RP-3, and Jet-A are the three most widely used hydrocarbon fuels in the aviation sector. Table 4 compares the basic properties of these hydrocarbon fuels, while Figure 10 presents their comprehensive performance differences. They exhibit significant differences in composition, physicochemical properties, and practical performance; detailed information is provided in Table 5. JP-10 (exo-tetrahydrodicyclopentadiene) is a high-density hydrocarbon fuel that, due to its excellent comprehensive performance, has been widely used in ramjet and scramjet engines [91,92]. Due to its unique cyclic structure, JP-10 possesses a high energy density, making it particularly suitable for aerospace applications with stringent volume and weight constraints, such as its use as fuel in scramjet engines to enable high-Mach-number flight [93,94]. However, JP-10 also has significant drawbacks, such as difficulty in ignition, low combustion efficiency, and high emissions of pollutants during combustion. To address these issues, researchers have undertaken efforts to improve the fuel by blending it with highly volatile substances such as propylene oxide (PO) [95] or diethyl ether (DEE) [96,97] to enhance its evaporation and combustion characteristics. RP-3 is a representative refined kerosene-type fuel in China. RP-3, as a typical representative of China’s refined kerosene-based aviation fuels, exhibits superior resistance to oxidation and coking under high-temperature conditions. This characteristic is crucial for the thermal stability of fuel in the regenerative cooling systems of supersonic aircraft [98,99,100]. At the same time, the vapor concentration of RP-3 fuel is a key parameter for assessing the flammability of aircraft fuel tanks [101]. Researchers have confirmed that RP-3 can be blended with diesel and used as an alternative fuel for diesel engines, and its spray combustion characteristics have been systematically and thoroughly studied [102]. Jet-A is a commonly used aviation fuel for turbofan engines; in-depth research into its combustion characteristics and comparisons with the performance of alternative fuels provide important guidance for engine design optimization and stable operation [103].

### 5.2. Mechanisms for Optimizing the Performance of Cluster Materials in Hydrocarbon Fuels

In recent years, researchers have incorporated nanoscale cluster materials into hydrocarbon fuels, a practice that has evolved into an effective technical approach for significantly enhancing the overall performance of fuels. The effects of these cluster materials can manifest in various ways, such as improving combustion efficiency, improving thermal stability, improving ignition performance, and reducing emissions, which will have far-reaching engineering prospects.

#### 5.2.1. Improving the Combustion Efficiency

In the early stage of combustion, aluminum clusters act as catalysts; they possess unique quantum size effects and high surface activity, which can influence the chemical pathways of combustion [118]. Laraib et al. reported that Al_15_ and Al_13_ clusters exhibited excellent adsorption properties for NO_2_ and SO_2_ [119], which indicates their potential in catalyzing hydrocarbon cracking and oxidation reactions. By lowering the activation energy of the reaction and accelerating chain reactions, aluminum clusters can promote the combustion process of hydrocarbon fuels at the molecular level. As the combustion progresses, these initially formed aluminum clusters will gradually grow through mechanisms such as further collisions, agglomeration, and surface adsorption and growth, eventually evolving into larger nAl [118]. nAl particles added to JP-10 fuel can significantly improve the combustion performance of the fuel and the efficiency of energy release. Compared with micron-sized aluminum particles, nAl particle fuel reduces ignition delay time [91,120]. Shen et al. [91] found that the addition of 1.0 wt% nAl particles improves the energy release efficiency of the JP-10 suspension fuel. In scramjet engines, JP-10 gel fuel containing 16 wt% nAl particles maintains stable combustion within a fuel equivalence ratio range of 0.6 to 1.0 under conditions of Mach 2 and a total temperature of 1700 K [92,121]. This performance improvement is primarily attributed to the larger specific surface area and higher chemical reactivity of the nAl particles, which accelerate the combustion process [104]. The addition of nAl particles also enhances the fuel’s heat absorption capacity, which is of significant importance for improving the performance of hypersonic aircraft fuels [122].

**Figure 11:**
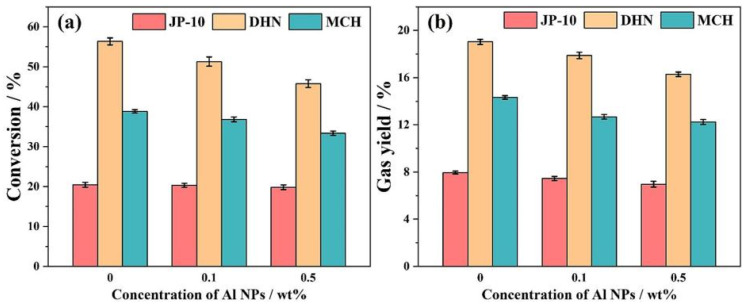
(**a**) Thermal cracking conversion and (**b**) gas yield of nanofluid fuels. Reprinted with permission from ref. [122]. © 2023 Elsevier B.V. All rights reserved.

Figure 11 visually illustrates the trends in cracking conversion rates (a) and gas yields (b) for the three fuels, decahydronaphthalene (DHN), methylcyclohexane (MCH), and JP-10, under different Al NP concentrations. The figure clearly shows that for DHN and MCH, both the conversion rate and gas yield decrease as the Al NP concentration increases. In contrast, these two parameters for JP-10 show no significant change, indicating that JP-10 is less affected by the inhibitory effect of Al NPs [122].

Zeolite catalysts can effectively improve the catalytic combustion properties of fuels containing aluminum nanoparticles. Among these, the nanolayer MFI zeolite (ZSM-5) acts as a catalyst by regulating pore structure, acidic sites, and layer thickness to selectively adsorb molecules of specific sizes, shapes, and polarities. Consequently, the catalytic combustion performance of fuels such as JP-10 can be enhanced [123].

**Figure 12:**
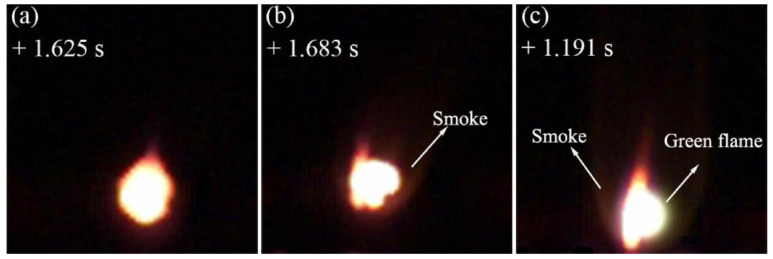
Combustion of agglomerates, (**a**) Micro-B/JP-10, (**b**) Nano-B/JP-10, (**c**) AlMgB/JP-10. Reprinted with permission from ref. [126]. © 2023 Elsevier B.V. All rights reserved.

Gupta et al. [124] reported that in a swirl-stabilized burner, the outlet temperature of a 10 wt% boron/JetA-1 slurry fuel was 19% higher than that of pure JetA-1. XRD and TGA analyses confirmed that the boron particles were completely oxidized—a finding of particular significance, as incomplete combustion of boron has long been a key bottleneck in this field. Jin et al. [125] reported that in supersonic ramjet combustion, JP-10 + 16 wt% B gel fuel achieved a 3.51% increase in combustion efficiency and an 8.42% increase in specific impulse compared to pure JP-10 gel fuel; however, they also found that boron particles resulted in an approximately 22% increase in heat flux, implying that the improvement in combustion efficiency comes at the cost of increased thermal protection requirements. By subjecting boron particles to nanoscale processing and alloying them with aluminum and magnesium, the engine can significantly improve the combustion efficiency of B/JP-10 suspension fuel. Nanoscale boron particles optimize the micro-explosive characteristics of fuel droplets, which shorten combustion time and accelerate the reaction process between boron and oxygen. Through interfacial reaction, the aluminum–magnesium–boron alloy reduces the activation temperature of boron, thereby improving the combustion reaction of boron and achieving high flame temperature and strong emission spectrum. Ultimately, the reaction rate between boron and oxygen is significantly increased, achieving more efficient energy release [126]. As demonstrated in Figure 12, which shows the combustion of agglomerates for Micro-B/JP-10, Nano-B/JP-10, and AlMgB/JP-10.

Boron’s high melting point, high boiling point, and surface oxide layer severely limit its combustion efficiency. In recent years, two innovative approaches have emerged, as shown in Table 6. The common logic underlying these approaches is that, rather than altering the properties of boron itself, it is more effective to indirectly improve combustion efficiency by modifying the combustion microenvironment, for example, by optimizing oxygen supply conditions or improving particle dispersion.

#### 5.2.2. Improving Thermal Stability

Nanofluid fuels containing aluminum nanoparticles—such as 0.1 wt% and 0.5 wt% aluminum nanoparticles dispersed in JP-10, DHN, and MCH—combine high energy density with heat absorption capabilities and hold promise for improving the flight performance of hypersonic aircraft [122]. Thermogravimetric analysis coupled with mass spectrometry (TGA-MS) is a key characterization method for evaluating the thermal cracking performance of such fuels. The addition of nAl particles to JP-10 improves the fuel’s thermal cracking performance; in particular, the addition of 0.1 wt% nAl particles to JP-10 yields the most significant thermal cracking effect [122].

**Figure 13:**
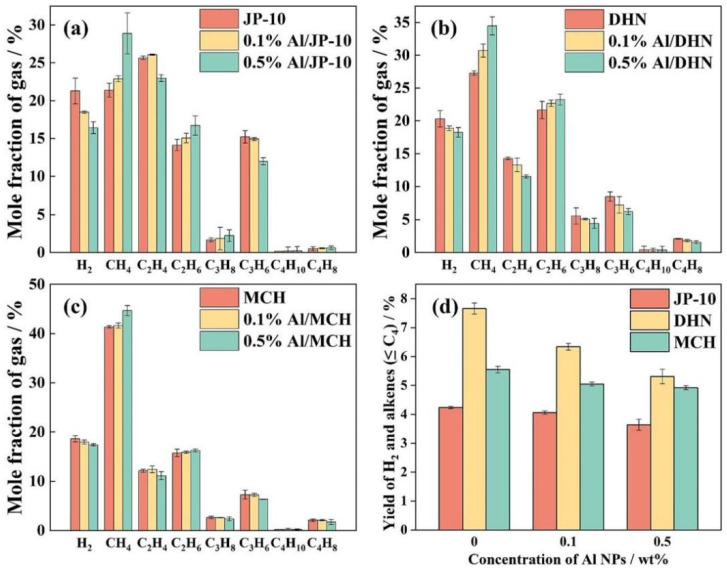
(**a**–**c**) Mole fraction of the major gaseous products and (**d**) yields of H_2_ and alkenes (≤C4) of thermal cracking of nanofluid fuels. Reprinted with permission from ref. [122]. © 2023 Elsevier B.V. All rights reserved.

Figure 13 shows the relative abundances of gaseous products resulting from the cracking of JP-10, DHN, and MCH nanofluidic fuels under the same heating procedure. It can be observed that methane constitutes the largest proportion of the MCH pyrolysis products, accounting for approximately 45 mol% of the gaseous products, whereas JP-10 and DHN produce higher proportions of ethylene, ethane, and propylene [122].

Wang et al. [129] reported that the atomically precise metal clusters [Ag_14_(C_4_B_10_H_11_)_12_(CH_3_CN)_2_] (CBA-Ag) and [Cu_6_Ag_8_(C_4_B_10_H_11_)_12_Cl] (CBA-CuAg), both protected by a C-borane, remain stable at temperatures above 200 °C. This thermal stability is of great importance for propellants; the material must remain inert within the storage and transport temperature range and be activated only upon contact with the oxidizer. Jiao et al. [130] synthesized [B_12_H_12_]^2−^, [B_10_H_10_]^2−^, and [B_6_H_7_]^−^ series salts, which exhibited excellent hydrolytic stability and thermal decomposition temperatures (Td) exceeding 200 °C. Water stability is particularly critical under practical storage and transportation conditions, providing the fundamental prerequisites for the large-scale application of these materials.

#### 5.2.3. Improving Ignition Performance

Blending JP-10 with DEE can improve the ignition sensitivity of JP-10 and reduce the concentration of incomplete combustion products [96]. Li et al. [131] demonstrated through their research that, regarding the propagation and evolution characteristics of JP-10/PO binary fuel in large-diameter pipelines, propylene oxide can effectively optimize the evaporation, combustion, and pollutant emissions of JP-10. Adding hyperbranched polyester (HPE) to JP-10 droplets promotes fuel evaporation and spontaneous combustion; bubbling and micro-explosions were observed, accelerating the evaporation and spontaneous combustion processes [132].

**Figure 14:**
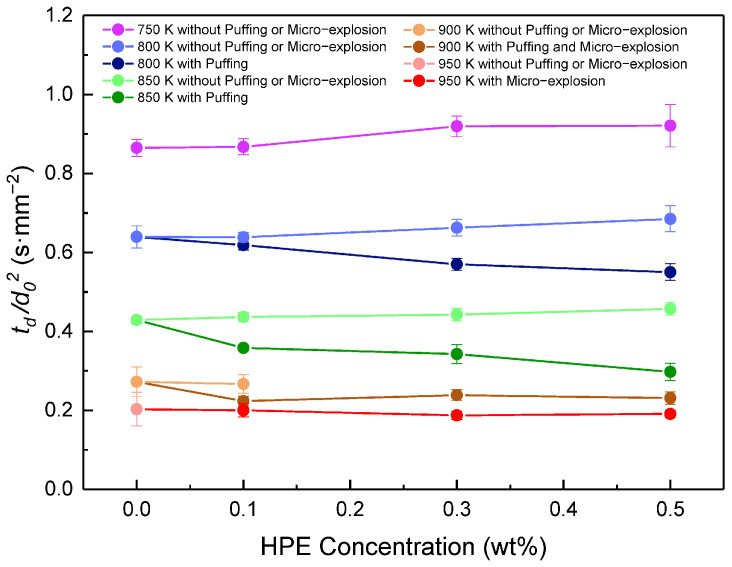
Variation in droplet lifetime with HPE concentration at temperatures of 750–950 K. Error bars show the standard deviation. Reprinted from ref. [132].

Figure 14 shows that during the evaporation process at 850 K, the addition of 0.1%, 0.3%, and 0.5% HPE reduces droplet lifetime by 16.5%, 20.1%, and 30.6%, respectively; during the self-ignition process at 900 K, the same concentrations of HPE reduce droplet lifetime by 18.0%, 12.4%, and 15.2%, respectively. Furthermore, HPE induces droplet expansion at 800 K and triggers micro-explosions in droplet mixtures containing 0.3% and 0.5% HPE at 900 K, further enhancing combustion efficiency [132].

Using ReaxFF molecular dynamics simulations, Wu et al. [133] revealed the atomic-level mechanism by which aluminum nanoparticles enhance methane combustion. The addition of aluminum nanoparticles significantly reduced the activation energy for methane dissociation by approximately 47%. It can be seen that the fundamental reason is that there is an abundance of low-coordinated atoms on the surface of Al_x_O_y_ clusters produced by the oxidation of aluminum nanoparticles. The chemical instability of the low-coordinated atoms leads to massive generation of atomic oxygen, and it is this highly reactive atomic oxygen that contributes more than 60% to the methane decomposition reaction. Interestingly, it was also found that even the aluminum particles completely covered by an oxide layer contributed, to some extent, to promoting the generation of atomic oxygen, and effectively enhanced the combustion of hydrocarbon fuels.

#### 5.2.4. Reducing the Ignition Delay Time

The IDT of CBA-CuAg was significantly shorter than that of CBA-Ag, indicating that hetero-metal doping can effectively accelerate the catalytic combustion process [129]. Copper doping is believed to further enhance the catalytic activity of cluster nuclei, thereby accelerating the reaction between ligands and oxidants. This observation offers a new way for the precise control of the fuel’s ignition response by alloying [134]. On the direct-connect platform of a scramjet engine, the IDT of 16 wt% Al NP-doped JP-10 gel fuel was much lower than that of pure JP-10 gel fuel [92]. The above result also proves that under actual engine operating conditions, nanoscale metal additives can reduce the IDT. As summarized in Table 7, which lists the ignition delay times for different cluster or nanoparticle material systems.

**Figure 15:**
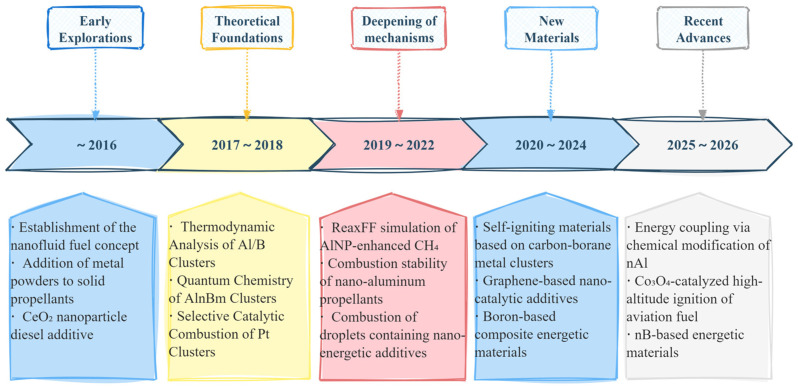
Timeline of the reinforcing effects of aggregated materials in the combustion of hydrocarbon fuels.

As shown in Figure 15, the timeline illustrates the retarding effects of aggregated materials in the combustion of hydrocarbon fuels. Based on precise control of the size, composition and carrier interactions of cluster materials, the catalytic performance can be optimally improved, the fuel combustion efficiency and energy conversion efficiency increased, and a feasible route developed for the development of new high-energy fuels and highly efficient hydrogen storage materials.

## 6. Challenges and Outlooks

Nanoclusters have promising application potential in hydrocarbon fuels, but the use of this application is still facing many critical challenges. These challenges include the long-term stability of nanoclusters in the fuel, large-scale production costs, the impact of combustion products on engines, and the development of multifunctional composite clusters. It is therefore necessary to focus on future research in the following areas: exploiting synergies between components, using molecular simulations to optimize the formulation, and breakthrough developments in high-efficiency and low-cost preparation technologies. Only by addressing these challenges one by one can nanoclusters be expected to be truly utilized in advanced fuels.

### 6.1. Balancing Long-Term Dispersion Stability and Fuel Performance

Achieving long-term stable dispersion of nanoclusters in hydrocarbon fuels is, in itself, a major challenge for engineering applications. Existing research has confirmed that nanoparticles typically require high concentrations of surfactants to achieve uniform dispersion in liquid fuels [135,136]. However, excessively high surfactant concentrations reduce the fuel’s evaporation rate, thereby affecting combustion performance. Yang et al.’s [135] studies on aluminum nanoparticles with varying degrees of oxidation indicate that unoxidized nanoparticles exhibit poor dispersion in JP-10, while partially oxidized nanoparticles demonstrate better stability, albeit with reduced ignition and combustion performance. This implies the need to find an appropriate balance between the degree of oxidation and combustion performance [137].

### 6.2. Effects of Combustion Products on the Engine

Solid oxides generated by the combustion of nanoclusters tend to form deposits within the engine. These deposits may cause engine wear or blockage of passages. In particular, when aluminum nanoparticles are added to JP-10 fuel, although the combustion temperature increases, the accumulation of solid deposits on the engine walls also increases, thereby affecting heat transfer characteristics [122]. Therefore, future research should focus on controlling the formation and deposition of combustion products to reduce potential damage to the engine.

### 6.3. Development of Multifunctional Composite Clusters

Bimetallic and multifunctional clusters with synergistic effects should be the focus of future research, as they can simultaneously solve the energy density, ignition performance, and carbon deposit shortcomings of existing fuel additives by combining the advantages of different materials. Research on the synergistic effect between different metal atoms can promote the optimization of catalytic performance. Metal nanoclusters with atomic precision have attracted considerable attention in fields such as electrocatalysis. The unique geometric and electronic structures of metal nanoclusters provide multiple active sites for adsorption and conversion reactions. Currently, techniques for synthesizing and characterizing sub-nanoscale metal clusters are continuously advancing, and their properties—such as fluxing behavior and reversible oxidation—are being thoroughly elucidated. These developments provide both theoretical and technical support for the research and development of multifunctional composite nanoclusters.

### 6.4. Optimization Aided by Molecular Simulation

Through molecular dynamics (MD) and quantum chemistry (QC) simulation, we can understand the behavior of nanoclusters in fuels in more detail, and then optimize the combustion performance. Zhou et al. [138] used ReaxFF and DFT to study the influence of electric fields on the pyrolysis of JP-10 fuel and its mechanisms, and fully proved the superiority of computational simulation in studying the microscopic reaction mechanisms of fuels. Through simulation, it is possible to predict the structure, energy characteristics, and nanomechanical properties of clusters, and even reveal the physicochemical properties and structural evolution patterns of materials at the atomic level.

### 6.5. Breakthroughs in High-Efficiency, Low-Cost Manufacturing Technologies

The large-scale application of nanoclusters is inseparable from efficient and economical preparation technologies. At present, nanoparticle preparation still faces many problems, such as poor reproducibility, low safety, high cost, and production difficulties. Therefore, it is urgent to develop preparation methods for high output, uniform particle size, and good stability. With the development and upgrading of technology, nanoclusters will play a more and more important role in high-energy-density fuels and advanced combustion technology.

## Figures and Tables

**Table 1:** Comparison of comprehensive properties of hydrocarbon-based modified materials.

Rating *	Category	A Typical Example	Principle	Key Advantages	Key Challenges
★☆ ☆☆	Oxygen-containing additive	Methanol, ethanol, MTBE	Provides additional oxygen atoms to promote complete combustion	Improves octane rating and reduces CO and hydrocarbon emissions	Low energy density; corrodes engine materials; has limited effectiveness in reducing ignition delay at low temperatures or under high pressure
★☆ ☆☆	Metal–organic compounds	Ferrocene, cerium naphthenate	Releases metal atoms during combustion and catalyzes radical reactions	As a combustion catalyst, it can theoretically accelerate the reaction	It tends to produce metal oxide ash, which leads to carbon buildup, fouling of spark plugs, and increased particulate emissions
★★ ☆☆	Conventional nanoparticles	Nano-aluminum (nAl), nano-boron, nano-copper oxide	Localized hot spots form due to the exothermic reaction caused by intense surface oxidation	High energy density and strong catalytic activity	Poor dispersion stability and a tendency to agglomerate; significant safety hazards, such as the risk of dust explosions; and low atomic utilization within the material
★★ ★☆	Cluster materials	Aluminum clusters, gold clusters, platinum clusters	Quantum confinement effects; extremely high surface-to-volume ratio; efficient activation of oxygen molecules	Extremely small particle size and excellent dispersion; low ignition threshold; extremely high catalytic efficiency; significantly reduced ignition delay	Difficult to produce on a large scale, poor thermal stability, and prone to agglomeration and deactivation

* The performance of different types of additives was compared and evaluated. The greater the number of star-shaped structures, the better the performance of the additive.

**Table 2:** The morphology, carrier and performance of metal cluster catalysts.

Metal Cluster Type		Metal Cluster Morphology	Carrier Material	Properties of Metal Clusters
Precious metal clusters	Pt	From single atoms to nanoparticles with a size of 1.40 nm [38]	CeO_2_, Fe_2_O_3_ [39,40]	A Pt monometallic catalyst was constructed through a dual nano-space confinement strategy, featuring abundant lattice oxygen and efficient elimination of VOCs [39]. Electronic structure and dispersion are the key factors influencing the catalytic performance [40]. The doping of Pt on Au_25_ clusters can form monometallic active sites [38].
	Pd	Nano-cluster	SBA-15 [41]	High thermal stability can be achieved through organophosphorus coordination anchoring, and it is not prone to agglomeration or loss [41].
	Rh	nano-particles	NiCo ferrite [42]	The particle distribution is more uniform, the surface area is higher, and the catalytic performance is excellent [42].
Transition metal clusters	Cu-Mn	-(Cu-Mn/Al_2_O_3_) [43]; Cu-MnO_2_ [44]	Al_2_O_3_ [43]; MnO_2_ [44]	Cu-Mn/A_l2_O_3_ exhibits high activity in toluene combustion (T90 = 260 °C, T50 = 237 °C), and the preparation method has a significant impact [43]; doping Cu into MnO_2_ can increase the specific surface area and oxygen vacancies, thereby enhancing the catalytic performance [44].
	Fe-Mn	Nano-crystals	Is itself an oxide.	Multiple VOCs exhibit catalytic activity at moderate temperatures. For instance, ethanol begins to undergo conversion at 230 °C and reaches approximately 97% at 300 °C [45].
	Co	Double perovskite structure	Uses itself as the framework	These double perovskites were controllably etched by nitric acid to remove part of the surface A-site and improve their catalytic activity for catalytic combustion of CB, toluene and benzene [46].
	Ni	Nano-composite materials [47]; Nin clusters (n = 1–6) [48]	Co_3_O_4_-ZrO_2_ (CZ) [47]; g-C_3_N_4_ [48]	Ni and La impregnation and substitution of Co_3_O_4_-ZrO_2_ achieved a 100% hydrogen combustion conversion rate below 350 °C [47]; Nin@g-C_3_N_4_ demonstrated structural stability and a favorable electronic structure [48].
	In	In_2_O_3_	ZrO_2_ [49]	As a potential alternative to traditional copper-based catalysts in the reaction of hydrogenation of carbon dioxide to methanol, the preparation method has a significant impact on its physical and chemical properties as well as catalytic performance [49].

**Table 3:** Common methods for cluster synthesis.

Method	Principle	Advantages	Limitations
Solvothermal/hydrothermal method	In a sealed, high-pressure solvent, the precursor forms clusters through slow reactions and self-assembly [65].	The conditions are mild and highly controllable, making this method suitable for the synthesis of ligand-protected metal clusters [66].	The reaction has a long duration, the mechanism is complex, and achieving reproducibility between batches poses a challenge.
Electrochemical synthesis method	By controlling the potential, metal electrodes are electrolysed in situ in the presence of ligands to form clusters [67].	It is highly economical, requires no external reducing agents, and allows for precise control of the oxidation state [5,67].	It requires sophisticated equipment, is difficult to mass-produce, and is limited to certain metal systems.
Gas-phase synthesis method	Clusters are formed and deposited in the vapor phase to create nanostructured films [68].	Enables the bottom-up synthesis of high-purity materials.	The yield is relatively low, the size distribution may be broad, and it is sensitive to energy parameters.
Template/Boundary Synthesis Method	Using porous materials such as MOFs and zeolites as templates to guide the nucleation and growth of clusters and suppress agglomeration [68,69,70,71,72].	The clusters are uniform in size, enabling monodisperse loading and controllable structure.	The process of removing the mold may damage the cluster structure, and post-processing is complex [73].
Wet Chemical Reduction Method	Metal salts are reduced in solution using reducing agents such as sodium borohydride or ascorbic acid, and nucleation is stabilized by ligands.	The reaction is fast and can be carried out at room temperature, making it suitable for the preparation of typical metal clusters such as gold and silver [74,75].	The size distribution may be broad, making agglomeration likely and making it difficult to precisely control the number of particles.

**Table 4:** Comparison of hydrocarbon fuel properties: JP-10, RP-3, Jet-A.

Features	JP-10	RP-3	Jet-A
components	Monocyclic hydrocarbon, exo-tetrahydro-dicyclopentadiene [104,105,106].	A highly refined paraffin-based blend consisting primarily of polycyclic and monocyclic naphthenes [107,108].	C8–C16 alkanes, cycloalkanes, and small amounts of aromatics (≤25% by volume) [109,110].
Density (g/cm^3^)	Approximately 0.93–0.94	Approximately 0.78–0.81	Approximately 0.77–0.80
Volumetric heat value (MJ/L)	Relatively high, at approximately 39.5	Somewhere between JP-10 and Jet-A	Lower, approximately 32.5–33.5
Freezing point	Excellent low-temperature performance [111].	Fairly good; suitable for aviation.	Excellent low-temperature flow properties [112].
Thermal stability	It is decent, but it may be prone to carbon buildup.	Refined and optimized for improved thermal stability [98].	Good
Applications	Supersonic aircraft, cruise missiles, ramjet engines [92,93,97].	Alternative fuels for military aviation and diesel engines [113].	Civil aviation

**Table 5:** Characterization techniques for cluster materials.

Characterization Techniques	Main Applications	Advantages	Limitations
Transmission Electron Microscope (TEM)	Morphology, size distribution, crystal structure, atomic arrangement; structural characterization of low-dimensional nanomaterials	High-resolution imaging at the atomic level; direct observation of crystal structures and defects; STEM provides Z-contrast images at the atomic scale, enabling the identification of active sites [114,115].	The equipment is complex; sample preparation is difficult; it has limited capabilities for analyzing amorphous materials; high-energy electron beams may damage sensitive samples
X-ray diffraction (XRD)	Crystal structure, phase composition, grain size; in situ monitoring of crystal structure evolution	Determine the crystalline phases and structure of materials; quantitatively analyze phase composition; in situ XRD enables real-time tracking of phase transformations.	It is not possible to directly identify amorphous or disordered cluster structures; analysis of complex mixtures is difficult; it is not sensitive to low-abundance phases
X-ray Photoelectron Spectroscopy (XPS)	Surface element composition, chemical state, oxidation state, and chemical bonding state	Surface-sensitive techniques provide information about the surface chemistry; they can distinguish between the valence states of elements and their chemical environments; and they allow for in-depth analysis.	Detects only a few nanometers deep into the surface; requires an ultra-high vacuum environment; limited sensitivity
X-ray Absorption Spectroscopy (XAS)	Local atomic structure, electronic structure, and valence state; in situ/operational tracking of dynamic changes in active sites	Characterizes the local coordination environment of elements; provides information on valence states and electronic structure; in situ/operational measurements enable real-time tracking of structural changes during reactions [116,117].	Requires synchrotron radiation, which is expensive and difficult to obtain; data processing and interpretation are complex; sensitivity to low-concentration samples is limited
Mass Spectrometry (MS)	Cluster molecular weight, chemical formula, exact atomic composition	Accurate determination of molecular weight and chemical formula; soft ionization techniques preserve the integrity of cluster structures.	Mild ionization should be used for clusters with poor thermal stability; analyzing complex mixtures poses significant challenges; spatial information cannot be provided

**Table 6:** New strategies for overcoming incomplete combustion of boron.

Strategy	Core Mechanism	Key Findings
Introduction of metal oxides (MOx) as solid oxygen carriers.	Compensate for insufficient oxygen supply during combustion.	TG-DSC confirms that the B-MOx system significantly enhances energy release [127].
Add ethanol (EtOH) to the B/JP-10 suspension fuel.	Promotes the fragmentation of boron aggregates, exposing a larger reaction surface area.	CO_2_ laser ignition experiments indicate that EtOH effectively promotes the fragmentation of aggregates and the release of energy [128].

**Table 7:** Comparison of ignition delay times for different cluster or nanoparticle material systems.

Materials System	Ignition Delay Time	Oxidizing Agent/Conditions
[B_12_H_12_]^2−^ and other borohydride salts [130]	As low as 1 ms	WFNA/N_2_O_4_
CBA-CuAg [129]	15 ms	White fuming nitric acid
CBA-Ag [129]	Self-ignites, but with a long ignition delay	White fuming nitric acid
Al NPs/JP-10 Gel [92]	Reduce ignition delay (qualitative)	Super-charged stamping Ma = 2

## Data Availability

No new data were created or analyzed in this study. Data sharing is not applicable to this article.

## Abbreviations

The following abbreviations are used in this manuscript:

PAHs	Polycyclic aromatic hydrocarbons
NCs	Nanoclusters
NPs	Ultrasmall nanoparticles
IDT	Ignition delay time
MIE	Minimum ignition energy
POMs	Polyoxometalates
MOFs	Metal–organic frameworks
MNCs	Metal nanoclusters
COFs	Covalent organic frameworks
SBUs	Secondary building units
MOS	Metal-oxide-semiconductor
TEM	Transmission electron microscope
XRD	X-ray diffraction
XPS	X-ray photoelectron spectroscopy
XAS	X-ray absorption spectroscopy
MS	Mass spectrometry
PO	Propylene oxide
DEE	Diethylene glycol monoethyl ether
DHN	Decahydronaphthalene
MCH	Methylcyclohexane
HPE	Hyperbranched polyester
MOx	Metal oxides
MD	Molecular dynamics
QC	Quantum chemistry
VOCs	Volatile organic compounds
